# Adaptive Dynamic Thresholds for Unsupervised Joint Anomaly Detection and Trend Prediction

**DOI:** 10.3390/s26010257

**Published:** 2025-12-31

**Authors:** Fenglin Ding, Yilin Zhao, Zongliang Li, Haibin Tang, Yizhuo Liu, Danhuai Guo

**Affiliations:** 1 Beijing Institute of Control Engineering, Beijing 100190, China; cast_ding@yahoo.com.cn (F.D.); zongnet@126.com (Z.L.); liuyizhuo98@outlook.com (Y.L.); 2 School of Astronautics, Beihang University, Beijing 100191, China; thb@buaa.edu.cn; 3 College of Information Science and Technology, Beijing University of Chemical Technology, Beijing 100013, China; zhaoyilin@buct.edu.cn

**Keywords:** anomaly detection, degradation prediction, adaptive threshold, prognostics health management (PHM), time series

## Abstract

Anomaly detection and degradation trend prediction are two pivotal tasks in system health management. However, most existing approaches treat them as independent problems and fail to exploit their intrinsic interdependence. In addition, the scarcity of labeled data in real-world scenarios limits the applicability of supervised learning methods. To address these challenges, we propose an adaptive thresholding strategy framework for unsupervised joint anomaly detection and trend prediction. Our framework introduces a self-adaptive threshold strategy from historical data distributions and dynamically updates them in response to evolving system behavior. The anomaly detection results are integrated to enhance degradation trend forecasting, while the predicted degradation trends, in turn, refine the anomaly thresholds through a feedback mechanism. Experiments on both public and real-world industrial datasets demonstrate that the proposed framework achieves superior detection accuracy, robust trend prediction, and high computational efficiency under diverse operational conditions.

## 1. Introduction

Ensuring the reliability and safety of complex engineering systems, such as aerospace propulsion, power generation, and energy infrastructures, requires effective health monitoring strategies. Early detection of abnormal behaviors and accurate prediction of degradation trends are two pivotal tasks in health management, as they enable timely recognition of system operating states, thereby preventing unexpected failures and reducing maintenance costs. However, the highly dynamic environments of such systems make robust health management a challenging task.

**Figure 1:**
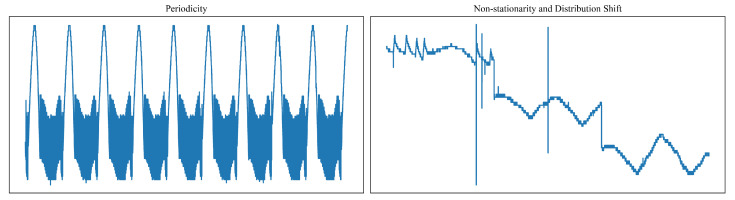
Periodicity, non-stationarity, and distributional drift in satellite telemetry data collected from onboard sensors.

In many real-world applications, the operational states of complex engineering systems are continuously monitored, and the resulting data are typically recorded as multivariate time series, which exhibit several key characteristics shown in Figure 1: (1) Periodicity [1]: Due to environmental influences or operational schedules, time series data from industrial systems often exhibit strong periodic patterns. These may manifest as seasonality, driven by external environmental cycles (e.g., daily, weekly, or annual repetitions), or as operation-mode periodicity, where specific parameters consistently change in a predictable manner during certain tasks (e.g., ignition of satellite thrusters). (2) Non-stationarity [2]: System data are influenced by complex and varying factors, making them inherently non-stationary. The relationships among variables can evolve over time, challenging methods that rely on stationary assumptions. (3) Distribution drift [3]: Time-series data may exhibit gradual distributional shifts over time. Such changes can result from evolving operating conditions, environmental influences, and degradation. Consequently, models that assume fixed data distributions or static thresholds may fail to detect emerging anomalies or accurately capture degradation trends.

Although several methods have employed adaptation techniques in anomaly detection models to address periodicity and distribution drift [4,5,6], the health management of complex systems continues to pose several key challenges: (1) Scarcity of labeled data [7]: In many real-world engineering systems, labeled data for degradation or failure events are often scarce or unavailable, making supervised learning approaches impractical. (2) Joint modeling of anomalies and degradation trends [8]: Anomalies and degradation trends are both crucial for health management and are inherently interrelated: short-term abnormal events can accelerate system degradation, while the underlying long-term degradation trend can influence the likelihood and severity of anomalies. Therefore, joint modeling of these two aspects is necessary for comprehensive and robust system monitoring. (3) Computational efficiency [9]: Health monitoring systems often generate large-scale, high-dimensional time-series data with stringent real-time requirements. Achieving high accuracy while maintaining computational efficiency remains a critical challenge for real-world health management applications.

To address these challenges, we propose a dynamic threshold framework for unsupervised joint anomaly detection and trend prediction. To tackle Challenge (1), we develop an adaptive thresholding method that generates thresholds from historical data, enabling unsupervised learning without relying on labeled anomalies. For Challenge (2), we investigate the interrelation between anomalies and degradation. Specifically, we design a degradation trend prediction method that leverages anomaly thresholding results, while simultaneously updating thresholds in response to the evolving degradation process. Regarding Challenge (3), the threshold-based method can significantly improve computational efficiency compared to heavy deep learning models, making it more suitable for real-time health management scenarios. Our main contributions can be summarized as follows:

- We develop an adaptive thresholding strategy that derives thresholds from historical data distributions while explicitly accounting for key characteristics of complex system time series, such as periodicity, nonstationarity, and distribution drift.
- We propose a unified framework that explicitly models the interactions between anomalies and degradation. Specifically, anomaly detection supports degradation trend prediction, while degradation modeling provides feedback to refine thresholds, enabling a mutually reinforcing loop for more robust health management.
- Our proposed framework demonstrates strong performance in real-world applications, providing accurate early warnings and robust trend predictions under limited labeled data scenarios.

## 2. Related Works

### 2.1. Threshold-Based Anomaly Detection

Threshold-based methods are among the most widely used techniques for anomaly detection in industrial systems, valued for their low computational cost, interpretability, and ease of deployment. In their simplest form, static thresholds are predefined using domain expertise or empirical statistical measures. Common approaches include expert defined thresholds [10] and standard deviation [11]. Although static thresholds are simple and effective under stationary conditions, they often fail to accommodate the dynamic nature of real-world data, resulting in high false alarm rates in practice.

To overcome these limitations, dynamic thresholding techniques have been introduced, which can adjust thresholds in real time based on the statistical distribution of recent data. Siffer et al. [12] proposed SPOT, a threshold-based approach that employs Extreme Value Theory to adaptively detect outliers in temporal data. To detect spacecraft anomalies, Hundman et al. [13] integrated LSTM models with a dynamic thresholding approach, enabling the method to effectively adapt to varying patterns in spacecraft data. In the context of gearbox anomaly detection, Dhiman et al. [14] applied adaptive thresholds with twin support vector machines. Ko et al. [15] employed the joint probability distribution of the ensemble denoising autoencoder’s outputs and corresponding residuals, introducing a variable threshold adaptively determined by confidence limits derived from the input variations. Recent studies have also explored threshold-based strategies in combination with advanced feature representation techniques. For example, Zhang et al. [16] employed VAE-GAN to learn feature representations and subsequently selected anomaly thresholds for effective detection of energy theft. Yang et al. [17] proposed an agent-based dynamic thresholding (ADT) framework that combines auto-encoder-based anomaly scoring with deep reinforcement learning to adaptively adjust thresholds for time series anomaly detection.

### 2.2. Degradation Trend Modeling and Prediction

In addition to point-wise anomaly detection, degradation modeling plays a critical role in capturing the long-term health evolution of complex systems. Early studies primarily employed stochastic process models such as Wiener processes [18] and Gamma processes [19] to describe gradual degradation. With the growing availability of sensor data, data-driven methods have become increasingly popular owing to their robustness and effectiveness. For instance, Zhang et al. [20] utilized a Kalman filter in conjunction with a BiLSTM model to predict the degradation trend of lithium-ion batteries. Chen et al. [21] applied a GRU-attention model on extracted performance degradation index for effective prediction of degradation trends. Ou et al. [22] leveraged an adaptive degradation stage characterization strategy and a distribution discrepancy evaluation gated recurrent unit (DDE-GRU) module to improve prediction accuracy. More recently, studies have combined physics-based and data-driven approaches to achieve more accurate degradation prediction. PI-LSTM [23] combined a physics-based calendar and cycle aging (CCA) model with an LSTM layer. Xu et al. [24] proposed a sequence-to-sequence deep learning framework with physical features to accurately predict battery capacity degradation trajectories from early-cycle data under various aging conditions. Despite these advancements, most existing methods still suffer from limited interpretability, which can undermine trust and complicate decision-making in critical applications.

### 2.3. Joint Anomaly Detection and Prediction

Typically, anomaly detection and degradation prediction have been treated as two separate problems. Anomaly detection methods focus on identifying short-term abnormal events, while degradation models aim to capture long-term health trends. In recent years, there has been growing interest in integrating these two tasks within a unified framework. Gomes et al. [25] designed a degradation indicator based on statistical anomaly detection results. Dimitrievska et al. [26] investigated both anomaly detection and trend prediction methods using photovoltaic plant data. Florkowski [27] applied image processing, machine learning, and optical flow techniques for joint anomaly detection and trend prediction in partial discharge patterns. Chen et al. [28] proposed a joint model for IT operation series prediction and anomaly detection, achieving superior performance on both tasks.

### 2.4. Summary and Limitations

Collectively, prior research has made significant progress in threshold-based anomaly detection and degradation trend prediction. However, static thresholds suffer from poor adaptability, degradation prediction methods offer insights into long-term health evolution but typically require supervised learning. Moreover, only a limited number of studies consider these two pivotal aspects in an integrated framework. Our proposed framework bridges this gap by incorporating thresholding into trend prediction, offering a novel perspective on health management.

## 3. Methodology

**Figure 2:**
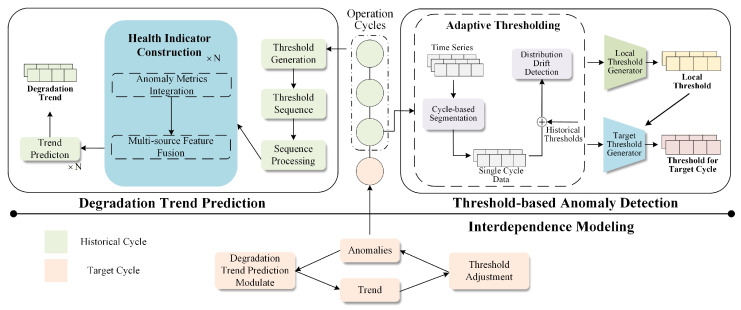
Overview of the proposed framework. The approach consists of two primary components: a Degradation Trend Prediction module, which forecasts the future evolution of the system’s health based on statistical features and the constructed degradation index (DI); and a Threshold-based Anomaly Detection module, which incorporates historical observations and the predicted degradation trend to generate dynamic thresholds for anomaly identification. Raw telemetry data are segmented into operational cycles, after which dynamic thresholds are iteratively updated using anomaly outcomes and predicted trends. Cycle-level statistics form a degradation index (DI), whose future trajectory is forecasted and fed back into the next threshold update.

This section introduces our proposed framework for unsupervised joint anomaly detection and trend prediction. As illustrated in Figure 2, our dynamic threshold-based framework consists of two primary components: (1) an adaptive thresholding module, and (2) a degradation trend prediction module.

The adaptive thresholding module generates thresholds for anomaly detection by capturing recurring patterns and trends from historical operational cycles. The data is initially segmented on the basis of cyclic operational patterns. The thresholds from the preceding segments are then used to compute a confidence score, which represents the proportion of points exceeding the threshold, indicating potential distributional changes. Local variation patterns are captured using sliding windows to generate new thresholds, minimizing the impact of high-frequency fluctuations and enabling more accurate threshold adaptation. These new thresholds are compared with previously fused thresholds and adaptively updated based on the confidence score. The updated thresholds are then applied to detect anomalies in the subsequent data segment.

The degradation trend prediction module is designed to estimate and forecast the long-term health trajectory of the system by leveraging both historical sensor data and anomaly information derived from the adaptive thresholding process. To achieve this, we employ a light weighted Transformer decoder-only model, which efficiently captures temporal dependencies and trends in the evolving system behavior while maintaining low computational complexity. By integrating anomaly scores, the module explicitly accounts for the interaction between short-term abnormal events and gradual system degradation, allowing it to anticipate the potential accelerated deterioration caused by recurring anomalies. This joint modeling approach not only enables robust and accurate predictions of future degradation trends but also provides feedback to adaptively refine anomaly detection thresholds, forming an interdependent mechanism that enhances overall system health monitoring.

The two modules interact in a mutually reinforcing manner: anomalies provide early indicators that inform degradation prediction, while the progression of degradation dynamically adjusts the anomaly thresholds. This synergistic approach improves the accuracy of the short-term anomaly detection accuracy and the robustness of long-term health monitoring.

### 3.1. Dynamic Thresholding for Anomaly Detection

To formulate an efficient method for online anomaly detection, we propose a dynamic thresholding approach that adapts to evolving data distributions and captures the underlying temporal patterns of the system. By leveraging historical operational cycles, the method continuously updates thresholds in response to changes in system behavior, enabling accurate detection of abnormal events without relying on labeled anomalies.

#### 3.1.1. Data Segmentation

Data segmentation is a critical step in adaptive thresholding. Unlike traditional seasonal data which are characterized by continuous periodic patterns, cycles in equipment data typically align with operational periods. Our design of segmentation rests on the assumption that, under stable environmental conditions and in the absence of faults, parameter variations within each operational cycle remain approximately consistent, and failures are unlikely to occur during non-operational intervals. Thus, cycle-based segmentation can be interpreted as distinguishing between operational and non-operational states.

In this study, K-means clustering is applied at the time-point level to multivariate sensor signals that reflect the instantaneous operational status of the equipment. The resulting clusters are interpreted as operational and non-operational states. Based on the temporal continuity of the point-wise clustering results, the time series is subsequently partitioned into cycles, which serves as a lightweight preprocessing step to support downstream thresholding and anomaly detection. Formally, for a time series *T*, segmentation decomposes it into a set of cycles S:

(1) $$S=\left\{S_{1},S_{2},\ldots ,S_{N}\right\}\in R^{N\times t}$$

where *N* denotes the number of segments and *t* is the length of each segment.

#### 3.1.2. Local Pattern Extraction and Threshold Generation

Existing dynamic thresholding methods typically rely on statistical measures of the data to compute and update thresholds. A common approach calculates the mean and standard deviation of a data segment and combines them into a single threshold, which is usually represented as a straight line. However, such thresholds often fail to account for the intrinsic fluctuations of the data and tend to be overly broad, resulting in a high false negative rate, particularly when the segment contains substantial internal variations. To overcome this limitation, we propose a method for extracting local thresholds that more closely tracks the underlying data dynamics. By capturing trend information, these local thresholds allow the model to learn finer-grained and more precise variation patterns during threshold fusion and update.

A sliding window is employed to capture local trends within each data segment. For each data segment, the sliding window traverses the sequence to generate local thresholds $t^{\mathrm{upper}}$ and $t^{\mathrm{lower}}$:

(2) $$t^{\mathrm{upper}}=\left\{x_{1}^{u},x_{2}^{u},\ldots ,x_{n}^{u}\right\}$$

(3) $$t^{\mathrm{lower}}=\left\{x_{1}^{d},x_{2}^{d},\ldots ,x_{n}^{d}\right\}$$

where $x_{i}^{u}$ and $x_{i}^{d}$ are the maximum and minimum values within the sliding window, adjusted by a parameter ϵ:

(4) $$x_{i}^{u}=\max\left(x_{i-k+1},x_{i-k+2},\ldots ,x_{i},\ldots ,x_{i+k}\right)+\epsilon$$

(5) $$x_{i}^{d}=\min\left(x_{i-k+1},x_{i-k+2},\ldots ,x_{i},\ldots ,x_{i+k}\right)-\epsilon$$

Here, *k* denotes the size of the sliding window. These local thresholds, extracted from each cycle of data, are subsequently fused in the dynamic threshold computation stage to form the final thresholds used for anomaly detection.

#### 3.1.3. Distribution Shift Detection

A key distinction between dynamic thresholds and manually set static thresholds lies in their ability to capture distributional changes within a cycle and autonomously recompute thresholds. Consequently, detecting such changes is a fundamental component of any dynamic thresholding algorithm.

Since a single data point is insufficient to characterize distributional shifts, and most real-world data deviate from normality, rendering the *z*-test inapplicable, we propose an alternative approach. Specifically, a peak threshold is first defined as the 95% of the anomaly threshold. Let *p* denote the proportion of points within a data cycle that exceed this threshold but are not classified as anomalies. Two parameters, $\delta_{1}$ and $\delta_{2}$, are subsequently introduced to guide the threshold update process:

(6) $$\Delta t=\left\{\begin{array}{cc} \mathrm{keep}, & p>\delta_{1},\\ \mathrm{partial}, & \delta_{2}<p\leq \delta_{1},\\ \mathrm{full}, & p\leq \delta_{2}, \end{array}\right.$$

This formulation enables the adaptive threshold to update based on the degree of distributional change.

#### 3.1.4. Dynamic Threshold Computation and Fusion

Unlike conventional dynamic thresholding methods that update thresholds only after detecting distributional changes, our approach updates thresholds at the end of each operational cycle, with the update strategy adapted to the degree of distributional shift. By using the entire cycle rather than individual data points as the update unit, the model can effectively captures subtle variations in normal patterns across different cycles.

After preprocessing and segmentation, the local thresholds $t^{\mathrm{upper}}$ and $t^{\mathrm{lower}}$ are extracted using Equations (4) and (5). Meanwhile, the distribution shift detection module quantifies the extent of the distributional change. These outputs are then combined to compute and update the dynamic thresholds for the subsequent cycle.

Let the current cycle be the *n*-th cycle in the data stream. Assume that the existing dynamic thresholds are derived from previous observations and the local thresholds extracted from the current cycle are denoted by $t^{\mathrm{upper}}$ and $t^{\mathrm{lower}}$. To enable smoother and more responsive adaptation to temporal variations, the anomaly thresholds for the next cycle, $t_{A}^{\mathrm{upper}}$ and $t_{A}^{\mathrm{lower}}$, are updated using an Exponential Weighted Moving Average (EWMA) [29] formulation:

(7) $$t_{A}^{\mathrm{upper}}\left(n\right)=\alpha \;t^{\mathrm{upper}}\left(n\right)+\left(1-\alpha \right)\;t_{A}^{\mathrm{upper}}\left(n-1\right)$$

(8) $$t_{A}^{\mathrm{lower}}\left(n\right)=\alpha \;t^{\mathrm{lower}}\left(n\right)+\left(1-\alpha \right)\;t_{A}^{\mathrm{lower}}\left(n-1\right)$$

where α∈(0,1) is the smoothing factor that determines the responsiveness of the update process. The dynamic threshold update procedure is shown in Algorithm 1.
**Algorithm 1:** Dynamic Threshold Update with Trend Forecasting Feedback
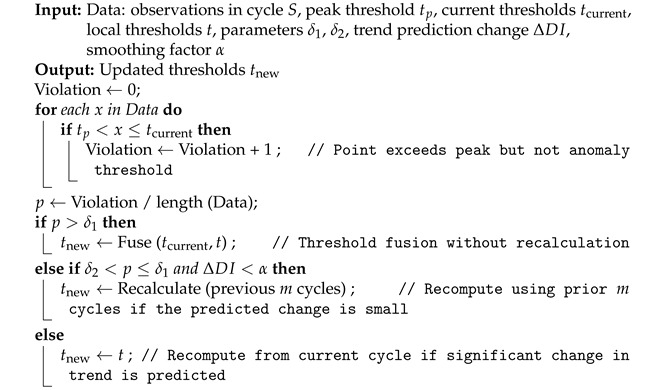


#### 3.1.5. Anomaly Detection

After the anomaly thresholds are computed, each data point can be classified as normal or abnormal based on its value. Specifically, a data point is considered normal when it lies between the upper threshold $t_{A}^{\mathrm{upper}}$ and lower threshold $t_{A}^{\mathrm{lower}}$. Conversely, it is regarded as abnormal if its value exceeds the upper threshold or falls below the lower threshold.

### 3.2. Degradation Trend Prediction

While anomaly thresholds focus on identifying short-term deviations within each operational cycle, degradation prediction aims to capture long-term performance decline across cycles. In practice, gradual degradation often manifests as subtle yet persistent shifts in the extracted local thresholds or anomaly frequencies, which cannot be fully explained by random fluctuations.

To this end, we model the temporal evolution of dynamic thresholds and anomaly indicators as a degradation signal. By applying statistical smoothing and trend extraction techniques, the model distinguishes between short-term noise and long-term deterioration patterns.

#### 3.2.1. Feature Construction

For each cycle *n*, we extract a feature vector $x_{n}$ that characterizes potential degradation. The construction of $x_{n}$ aims to capture both short-term fluctuations and long-term shifts in equipment behavior. Specifically, the following types of features are considered:

- Relative change in dynamic thresholds $\Delta_{t}^{\left(n\right)}$. The relative difference between the updated dynamic thresholds of the current cycle and those of the previous cycle reflects gradual drifts in the decision boundary. Such drifts often indicate the accumulation of wear or performance loss over time.
- Absolute shift of local thresholds ${\bar{\delta }}_{t}^{\left(n\right)}$. The absolute displacement of cycle-level local thresholds provides fine-grained information about variations in normal operating patterns. Persistent increases or decreases in these values can signal long-term deterioration that is not immediately detectable through anomaly counts alone.
- Proportion of points exceeding the peak threshold $p_{n}$. This feature quantifies the fraction of data points that surpass the peak threshold but are not classified as anomalies. A rising $p_{n}$ across cycles suggests that the baseline operating condition itself is shifting toward more extreme values, which is a typical symptom of degradation.
- Statistical descriptors of the cycle data. Standard metrics (mean $\mu_{n}$, variance $\sigma_{n}$, and quantiles $q_{n}$) capture distributional changes within the cycle. Increasing variance may indicate unstable operation, while systematic shifts in the mean or quantiles often reveal gradual decline in equipment health.

All extracted features are standardized to ensure comparability across dimensions and concatenated into a single vector:

(9) $$x_{n}=\left[\Delta_{t}^{\left(n\right)},{\bar{\delta }}_{t}^{\left(n\right)},p_{n},\mu_{n},\sigma_{n},q_{25,n},q_{75,n}\right],$$

#### 3.2.2. Degradation Index Computation

Once the feature vector $x_{n}$ is constructed, it is mapped into a scalar degradation index (DI) that summarizes the health condition of the equipment for cycle *n*. At this stage, we adopt a simple averaging strategy:

(10) $$DI_{n}=\frac{1}{d}\sum_{i=1}^{d}x_{n,i},$$

where *d* is the dimensionality of the feature vector $x_{n}$.

To reduce the effect of short-term fluctuations while retaining long-term degradation trends, the raw DI sequence is further smoothed using an Exponential Weighted Moving Average (EWMA):

(11) $${\tilde{D}I}_{n}=\lambda {\tilde{DI}}_{n-1}+\left(1-\lambda \right)DI_{n},$$

Here, λ controls the degree of smoothing, with higher values giving more weight to previous cycles. The smoothed degradation index ${\tilde{DI}}_{n}$ serves as the input for subsequent trend analysis and predictive modeling.

#### 3.2.3. Trend Forecasting

To ensure computational efficiency while retaining predictive accuracy, we adopt a lightweight forecasting approach based on Transformer decoder which contains only a single-layer multi-head attention block followed by a compact feed-forward module with residual and layer normalization. The attention block employs a local-window mechanism, which reduces the effective computational complexity from $O(L^{2})$ to approximately O(L·w), where *w* is the window size.

Formally, given a smoothed degradation index (DI) sequence $\left\{{DI}_{1},{DI}_{2},\ldots ,{DI}_{n}\right\}$, each element is first mapped into a *d*-dimensional embedding space and enhanced with positional embeddings to preserve temporal order. The decoder then takes the most recent *L* steps as input context and predicts the next step in an auto-regressive manner. At each decoding step *t*, the decoder attends to *w* previous representations $h_{t-w}^{\left(l-1\right)},\ldots ,h_{t-1}^{\left(l-1\right)}$ through a multi-head self-attention mechanism:

(12) $$A^{\left(l\right)}=\mathrm{softmax}\;\left(\frac{Q^{\left(l\right)}{\left(K^{\left(l\right)}\right)}^{⊤}}{\sqrt{d_{k}}}\right)V^{\left(l\right)},$$

The result of the attention layer is then passed through a feed-forward network (FFN) applied to each time step independently:

(13) $$\mathrm{FFN}\left(x\right)=\sigma \left(xW_{1}+b_{1}\right)W_{2}+b_{2},$$

where σ(·) is a nonlinear activation function. Finally, the output representation of the last decoder layer $O_{t}^{\left(L\right)}$ is mapped to the predicted degradation index:

(14) $${\hat{D}I}_{t}=O_{t}^{\left(L\right)}W_{out}+b_{out}.$$

The forecasting objective minimizes the mean squared error between the predicted and actual degradation indices over the horizon *H*:

(15) $$L_{trend}=\frac{1}{H}\sum_{h=1}^{H}{\left({\hat{D}I}_{n+h}-DI_{n+h}\right)}^{2}.$$

where ${\hat{D}I}_{n+h}$ denotes the predicted degradation index at the future time step n+h, $DI_{n+h}$ is the corresponding ground-truth degradation index.

### 3.3. Overall Workflow of the Proposed Framework

The proposed framework follows a unified workflow that integrates data preprocessing and segmentation, dynamic thresholding, degradation index construction, and trend prediction. As illustrated in Figure 2, the end-to-end process consists of four major stages.

(1) Data preprocessing and cycle-based segmentation. The raw telemetry data are first preprocessed and subsequently segmented into distinct operational cycles through a k-means clustering procedure.
(2) Dynamic thresholding and anomaly detection. The dynamic threshold is iteratively updated based on the anomaly detection outcome and the trend prediction results obtained in the previous round. An anomaly is flagged whenever the data exceeds the adaptive boundary.
(3) Degradation index construction. After the threshold is updated, the degradation index is constructed based on the cycle-level data and their corresponding thresholds.
(4) Trend prediction through sequence forecasting. A forecasting model is utilized to estimate the future trajectory of the degradation index (DI). The predicted trend serves as a reference baseline and is incorporated into the next iteration of dynamic threshold updates to enhance sensitivity to gradual degradation.

### 3.4. Interactions Between Modules

The interaction between the Trend Forecasting and Dynamic Thresholding modules is crucial for the framework’s adaptability and accuracy. As the Trend Forecasting module predicts the system’s future degradation trends based on historical data, these predictions inform the Dynamic Thresholding module by providing updated expectations for the system’s long-term behavior. Specifically, the forecasts from the Trend Forecasting module offer insights into whether the system’s degradation trajectory will remain stable or accelerate in the coming cycles. For example, when the system is predicted to experience more rapid degradation, the thresholds are modified to become more sensitive to smaller changes, ensuring timely anomaly detection.

By incorporating these predictions, the system can efficiently adapt to both gradual health declines and sudden shocks, maintaining high sensitivity to real-time anomalies while accounting for the underlying trends in the data.

## 4. Experiments

### 4.1. Experimental Setup

This section outlines the datasets, baseline methods, evaluation metrics, and implementation details used in our experiments.

#### 4.1.1. Datasets

**Figure 3:**
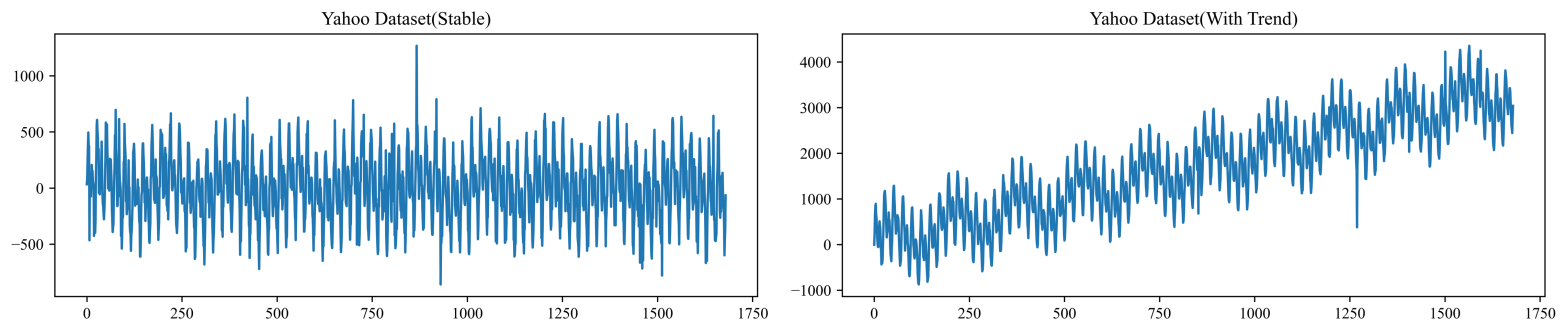
Sample series of Yahoo Dataset.

To evaluate the performance of our proposed framework across diverse scenarios, we conduct experiments on a public dataset and a real-world satellite thruster dataset. The Yahoo Webscope dataset [30] is a publicly available dataset released by Yahoo Labs. It contains 367 labeled time series, including both real and synthetic data, with each series consisting of 1420 to 1680 instances. The dataset includes stationary periodic series as well as series that exhibit periodicity combined with evolving trends. Table 1 summarizes the statistics of both datasets, while Figure 3 presents representative time-series examples from the Yahoo dataset. The satellite thruster dataset originates from real-time measurements of thrusters employed for orbit maintenance and attitude control in on-orbit satellites. It consists of multiple sensor readings, including current and voltage parameters, which capture the operational characteristics of the thrusters. Appendix A.2 provides additional details of the satellite thruster dataset.

#### 4.1.2. Baselines

We compare our proposed dynamic thresholding framework in the anomaly detection task against the following representative methods:

- OCSVM [31], an unsupervised anomaly detection algorithm that models the normal data distribution and identifies outliers as anomalies.
- Isolation Forest [32] uses random partitioning to recursively isolate data points.
- SPOT [12], an anomaly detection algorithm that leverages Extreme Value Theory (EVT) to detect outliers in time series data.
- DSPOT [12], an enhanced version of SPOT that adapts to changes in the data by dynamically adjusting the thresholds.
- SI-ATFD [33] combines stacked machine learning with adaptive residual thresholding to enable fast and accurate early fault diagnosis.
- AT2SD [34] proposes a two-stage threshold-based detector for DDoS detection.
- DONUT [35] applies variational autoencoders in unsupervised anomaly detection.
- TranAD [36], an unsupervised anomaly detection algorithm that leverages the Transformer architecture to capture long-range dependencies and detect anomalies in time-series data.
- Anomaly Transformer [37] proposes Anomaly Attention mechanism to model the prior association and the series association.

In terms of trend prediction, we compare our method with several representative approaches, including ARIMA [38], LSTM [39], TCN [40], CF-RUL [41] and BiLSTM-MHSA [42].

#### 4.1.3. Evaluation Metrics

To evaluate our model’s anomaly detection performance, we adopt Precision, Recall, and F1-score as the primary metrics.

(16) $$\mathrm{Precision}=\frac{TP}{TP+FP}$$

(17) $$\mathrm{Recall}=\frac{TP}{TP+FN}$$

(18) $$F1=\frac{2\times \mathrm{Precision}\times \mathrm{Recall}}{\mathrm{Precision}+\mathrm{Recall}}$$

where TP is the number of true positives, FP is the number of false positives and FN is the number of false negatives.

As for the prediction task, we employ Mean Squared Error (MSE), Root Mean Squared Error (RMSE) and Mean Absolute Error (MAE) in the evaluation:

(19) $$\mathrm{MSE}=\frac{1}{N}\sum_{i=1}^{N}{\left(y_{i}-{\hat{y}}_{i}\right)}^{2}$$

(20) $$\mathrm{RMSE}=\sqrt{\frac{1}{N}\sum_{i=1}^{N}{\left(y_{i}-{\hat{y}}_{i}\right)}^{2}}$$

(21) $$\mathrm{MAE}=\frac{1}{N}\sum_{i=1}^{N}\left|y_{i}-{\hat{y}}_{i}\right|$$

where *N* is the number of samples, $y_{i}$ is the ground truth value, and ${\hat{y}}_{i}$ is the predicted value.

#### 4.1.4. Implementation Details

Experiments were conducted on a Lenovo laptop (Lenovo Group Ltd., Beijing, China) equipped with a NVIDIA 3060 Laptop GPU (NVIDIA Corporation, Santa Clara, CA, USA) and an AMD Ryzen 7 5800H CPU (Advanced Micro Devices, Inc., Santa Clara, CA, USA). The implementation was developed in Python using NumPy (Version 2.1.3), scikit-learn (Version 1.5.2), and PyTorch (Version 2.5.1). GPU acceleration was enabled via CUDA (Version 12.4).

We employ the Adam optimizer with a learning rate of 0.001 for the trend prediction module. For the Yahoo dataset, a sliding window strategy with a fixed window size of 200 is adopted to construct cyclic input sequences. In the dynamic thresholding module, the sliding window size *k* is set to 40 for the Yahoo dataset and 100 for the Satellite dataset. The hyper-parameters $\delta_{1}$ and $\delta_{2}$ are set to 0.95 and 0.85 respectively for both datasets. All input time-series data are normalized using the StandardScaler, which standardizes each feature according to:

(22) $$z=\frac{x-\mu }{\sigma }$$

where *x* is the original feature value, μ is the mean of the feature in the training data, and σ is the standard deviation. More detailed parameter configurations are reported in Appendix A.1.

### 4.2. Overall Results

#### 4.2.1. Unsupervised Anomaly Detection

To validate the effectiveness of the proposed model, we conduct experiments on an unsupervised anomaly detection task. The results are presented in Table 2. To alleviate the bias introduced by point-wise evaluation on contiguous anomaly regions, we further adopt a delayed F1 metric in Appendix B.1 as well as a paired *t*-test in Appendix B.2. From the results, we observe:

- Traditional methods such as OCSVM and Isolation Forest exhibit unstable performance, as they rely on static decision boundaries that cannot adapt to the dynamic and periodic characteristics of the data.
- Methods based on Extreme Value Theory (EVT), including SPOT and DSPOT, achieve moderate improvements by introducing adaptive thresholds. However, they fail to capture periodic patterns in common industrial data and cannot effectively respond to abrupt changes, making them insufficient for anomaly detection on periodic data.
- Deep learning-based approaches DONUT, TranAD, and Anomaly Transformer show stronger adaptability by leveraging latent representations, yielding better precision recall trade-offs compared to traditional methods.
- Compared to other threshold-based PHM methods, our proposed model achieves the highest overall F1 scores, confirming its capability to handle periodic data and working cycles. By dynamically updating thresholds based on both recent data distributions and long-term trends, our model can maintain reliable detection even under shifting data distributions, outperforming all baseline methods in overall accuracy and stability in most industrial situations.

#### 4.2.2. Trend Prediction

**Figure 4:**
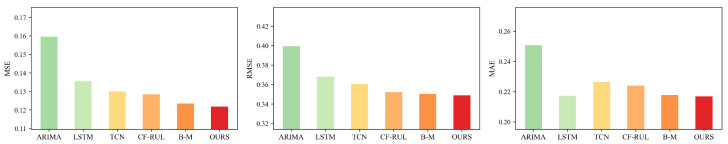
The prediction performance of our proposed framework in comparison to baselines.

The trend prediction performance is evaluated on both datasets, and the results are shown in Figure 4.

As illustrated in Figure 4, our method consistently outperforms the baselines across all evaluation metrics. Specifically, our model achieves the lowest MSE of 0.1217, indicating superior accuracy in trend prediction compared to ARIMA (0.1595), LSTM (0.1355), TCN (0.1299), CF-RUL (0.1284) and BiLSTM-MHSA (0.1236). These results suggest that our framework excels at capturing long-term dependencies and complex patterns in the data, and can effectively predict degradation trends in real industrial applications.

### 4.3. Computational Efficiency

To evaluate the computational efficiency of the proposed model, we analyze both runtime and memory consumption of the dynamic thresholding process compared with existing baseline methods. Since the trend forecasting module is executed independently and does not dominate the overall computation, our focus is placed on the anomaly detection phase, which is the primary component in real-time deployment. The experiments are performed on A3Benchmark, a subset of the Yahoo dataset containing 100 time-series samples, each comprising 1580 data points. The results are shown in Table 3.

Among all baselines, our proposed method achieves the lowest computational cost while maintaining stable performance. Specifically, our model requires only 10.94 s to process the entire dataset, which is approximately 1.1× faster than Isolation Forest, 3× faster than OCSVM, and more than 12× faster than SPOT. In terms of memory usage, our method also shows clear advantages, consuming only 339.86 MB. This improvement mainly stems from the lightweight design of the dynamic thresholding mechanism, which incrementally updates the upper and lower bounds without full historical recomputation.

### 4.4. Ablation Study

**Figure 5:**
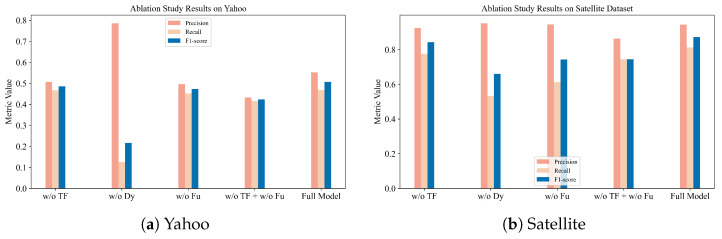
(**a**) Ablation study results on Yahoo dataset. (**b**) Ablation study results on Satellite dataset.

To better understand the contribution of each component in the proposed framework, we conduct a series of ablation experiments by removing them from the full model, which includes (1) w/o TF: removes the trend forecasting module, which captures long-term degradation tendencies; (2) w/o Dy: removes the dynamic threshold updating mechanism, which adaptively adjusts the upper and lower bounds; (3) w/o Fu: replaces the EWMA-based threshold fusion with a simple average when updating the anomaly thresholds. Results are shown in Figure 5 and the detailed results are provided in Appendix B.3.

It can be observed that removing any of the components leads to a noticeable degradation in overall performance. When the trend forecasting module (w/o TF) is removed, the model loses its ability to anticipate long-term degradation tendencies, resulting in delayed or missed anomaly detections. Similarly, eliminating the dynamic threshold updating mechanism (w/o Dy) greatly reduces adaptability to distributional shifts, resulting in increased missed detection. Moreover, replacing the EWMA-based fusion strategy with a simple averaging scheme (w/o Fu) makes the model more sensitive to short-term fluctuations and noise, leading to unstable detection performance. Notably, the performance degradation caused by jointly removing TF and Fu is larger than that observed when removing either component alone, indicating a non-additive interaction between trend forecasting and feature fusion.

### 4.5. Segmentation Stability

To verify robustness of K-means clustering, we conduct a sensitivity analysis based on the Adjusted Rand Index (ARI). Specifically, we inject additive noise and slow drift into the raw sensor signals and compute ARI between the original and perturbed cluster assignments.

Table 4 reports the ARI-based sensitivity analysis for point-wise operational state clustering. The clustering remains unchanged under additive Gaussian noise up to 10% of the signal standard deviation and bias drift up to 20%. These results demonstrate that the proposed point-wise clustering is highly stable and robust under realistic noise and drift conditions.

### 4.6. Parameter Sensitivity

**Figure 6:**
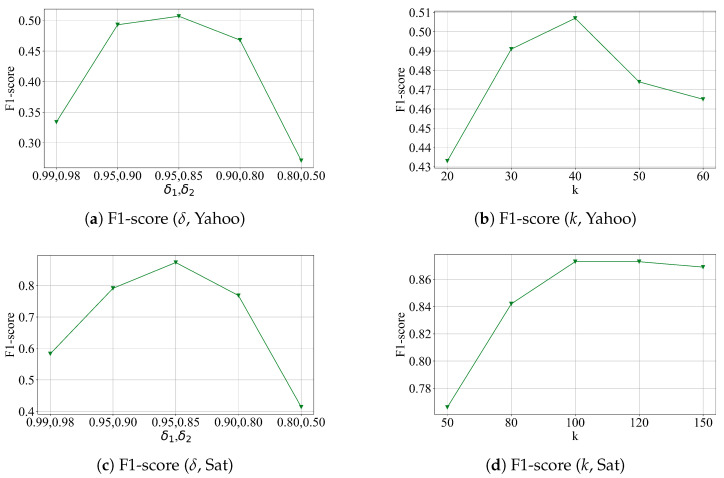
Parameter sensitivity tests of the model on two datasets.

We conduct experiments on the two datasets to study the impacts of the following hyper-parameters. $\delta_{1}$ and $\delta_{2}$ are parameters that control the threshold update, and *k* is the sliding window size in local pattern extraction and threshold generation module; the results are shown in Figure 6.

Figure 6a,c show the result of varying δ. The performance drop as we increase or decrease the threshold of distribution change detection. This is because a smaller δ causes the model to over-react to minor fluctuations, leading to frequent threshold updates and false alarms. In contrast, a larger δ makes the model less responsive to actual distribution shifts, resulting in delayed or missed anomaly detections.

Figure 6b,d illustrate the result of varying *k*. We observe that the performance drops when a smaller window size is selected, as the extracted patterns fail to capture sufficient temporal context and result in unstable feature representations.

### 4.7. Case Study

#### 4.7.1. Adaptive Threshold Result Comparison

**Figure 7:**
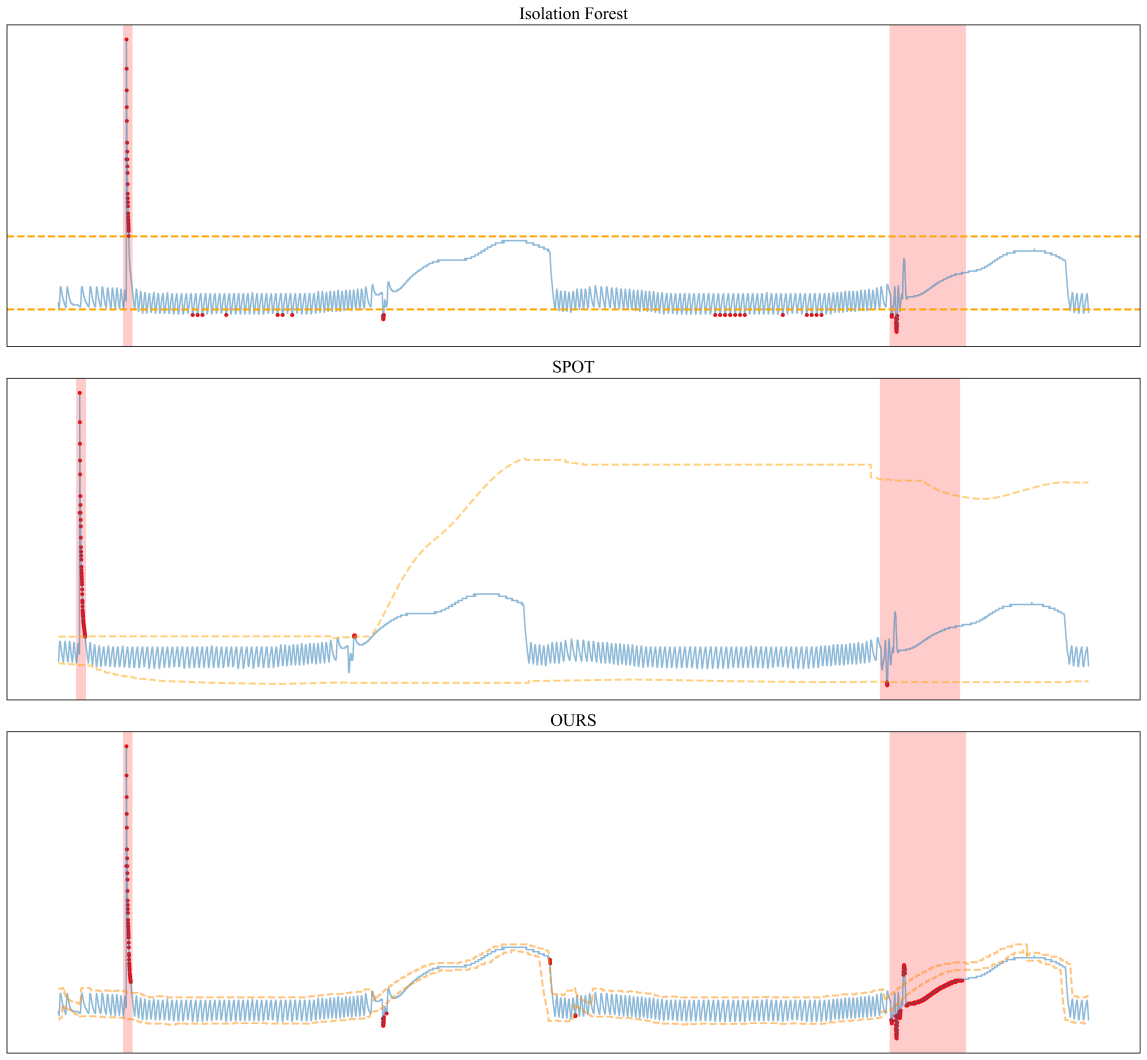
Case Study on the Satellite Thruster Dataset: The red background section indicates the true anomaly region, the blue curve represents the actual data, the upper and lower orange dashed lines denote the thresholds computed by the model, and the red dots mark the anomaly points detected by the model.

To verify the effectiveness and superiority of the proposed thresholding algorithm, we apply the proposed dynamic thresholding model to the satellite thruster dataset and compare its performance with two traditional thresholding methods under real anomaly cases. The results are shown in Figure 7.

The results demonstrate that the proposed method can accurately detect changes in the data distribution, dynamically compute, update, and fuse thresholds for anomaly detection, and successfully identify all actual fault occurrences. Moreover, the proposed model achieves better performance than the baseline methods, exhibiting higher detection accuracy and stronger adaptability to distributional variations.

As illustrated in the figure, the Isolation Forest algorithm determines anomalies solely based on distance metrics and outputs a static linear threshold. Such a method cannot effectively detect or recognize contextual anomalies, leading to missed detections in the second anomalous region. Furthermore, Isolation Forest separates anomalies according to a predefined contamination ratio, which often results in false positives.

In comparison, the SPOT algorithm generates thresholds that better align with the real data distribution and can adapt to gradual changes over time. However, SPOT fails to capture periodic patterns in the data and cannot effectively respond to abrupt changes. In some cases, it becomes overly sensitive to short-term fluctuations. As shown in the figure, after detecting an upward trend, SPOT recalculates thresholds based on the updated distribution, causing a substantial increase in the upper bound. Such excessively broad thresholds greatly reduce the algorithm’s sensitivity to anomalies, resulting in a high miss-detection rate.

Our proposed method performs notably better on periodic datasets. As shown in the results, the model effectively extracts periodic information and updates the thresholds according to cyclic variations. This approach produces dynamic thresholds that closely follow the true data distribution. Although some false alarms may occur, the algorithm achieves a significantly higher anomaly detection rate overall. In practical applications, the ability to detect all true anomalies is often of higher priority, a moderate increase in false positives is acceptable when it improves the overall reliability of detection.

#### 4.7.2. Temporal Evolution of the Adaptive Threshold

**Figure 8:**
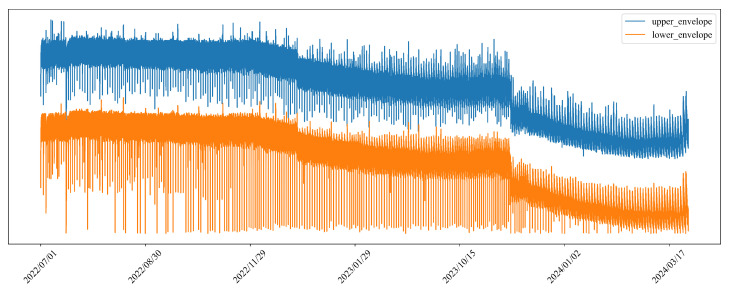
Temporal evolution of the dynamic upper and lower thresholds. The thresholds are updated iteratively and exhibit a gradual downward trend over time, reflecting adaptation to long-term performance degradation.

To further illustrate the effectiveness of the designed interaction between the adaptive thresholding mechanism and the trend prediction module, as well as the rationale behind the DI construction, we visualize the temporal evolution of the dynamic thresholds. As shown in Figure 8, the thresholds gradually shift over time rather than remaining static, indicating that the thresholding mechanism adapts to long-term degradation trends. This result demonstrates how the threshold-related DI and the associated trend prediction jointly guide the adaptive thresholding process, and further confirms that the selected features used for DI construction can effectively capture degradation trends.

## 5. Conclusions

In this paper, we propose a dynamic thresholding framework for unsupervised joint anomaly detection and trend prediction. The adaptive thresholding module derives anomaly thresholds from historical data distributions and incrementally updates them to form future thresholds, ensuring real-time adaptability to distributional shifts. Meanwhile, the trend prediction module employs a lightweight Transformer decoder and utilizes the constructed degradation index to forecast long-term degradation trends. The results from the trend prediction module are further utilized to assist in refining the dynamic thresholds, forming a feedback loop between trend forecasting and anomaly detection. This feedback mechanism enables the system to continuously calibrate the anomaly boundaries based on predicted degradation trends, thereby enhancing sensitivity to early-stage anomalies. Experiments on two large-scale datasets demonstrate the effectiveness of our proposed framework. However, two limitations remain: (1) the dynamic thresholding mechanism operates on a one-dimensional telemetry signal and therefore cannot explicitly model cross-variable interactions, such as time differentials associated with changes in multiple sensor channels; (2) the absence of RUL labels limits the extent to which the degradation trend prediction module can be utilized. With a DI constructed through simple aggregation, the forecasting module currently serves mainly as an auxiliary component for dynamic threshold refinement, instead of performing full prognostic tasks such as RUL estimation. In the future, we plan to explore the method of multi-dimensional thresholding, which will enable the model to jointly consider correlations across different variables when determining anomaly boundaries.

## Figures and Tables

**Table 1:** Statistics of the public dataset.

Category	Yahoo	Thruster
Series	367	600
Time-stamps	572,966	91,967,123
Anomalies/Events	3896 (0.68%)	501,752 (0.55%)
Granularity	Hourly	Half-secondly

**Table 2:** Comparison of unsupervised anomaly detection performance on Yahoo and Satellite datasets over 5 random seeds. ↑ indicates that higher values are better. **Bold** values indicate the best performance.

Method	Yahoo			Satellite		
	F1-Score ↑	Precision ↑	Recall ↑	F1-Score ↑	Precision ↑	Recall ↑
OCSVM	0.025±0.004	0.014±0.002	0.182±0.007	0.502±0.019	0.373±0.022	0.768±0.013
Isolation Forest	0.292±0.018	0.185±0.015	0.703±0.021	0.226±0.011	0.729±0.034	0.134±0.008
SPOT	0.424±0.012	0.379±0.018	0.481±0.009	0.611±0.012	0.964±0.007	0.447±0.011
DSPOT	0.322±0.007	0.253±0.009	0.443±0.010	0.651±0.010	0.969±0.013	0.490±0.011
SI-ATFD	0.374±0.007	0.315±0.009	0.459±0.010	0.618±0.015	0.726±0.018	0.539±0.026
AT2SD	0.431±0.010	0.378±0.013	0.501±0.007	0.587±0.006	0.664±0.005	0.526±0.006
DONUT	0.198±0.010	0.154±0.009	0.277±0.011	0.599±0.007	0.740±0.009	0.503±0.006
TranAD	0.433±0.004	0.401±0.007	0.470±0.005	0.756±0.005	0.831±0.006	0.693±0.006
Anomaly Transformer	0.273±0.001	0.224±0.005	0.352±0.009	0.738±0.002	0.807±0.003	0.681±0.002
OURS	0.509±0.002	0.555±0.002	0.470±0.003	0.873±0.000	0.944±0.000	0.812±0.001

**Table 3:** Runtime and memory consumption result on A3Benchmark. **Bold** values indicate the best performance.

Baselines	Runtime (s)	Memory (M)
OCSVM	32.47	379.29
Isolation Forest	11.96	424.09
SPOT	133.20	376.72
OURS	**10.94**	**339.86**

**Table 4:** Segmentation stability under noise and drift measured by ARI.

Perturbation Type	Level	ARI
Gaussian noise (η)	0.01	1.0000±0.0000
	0.05	1.0000±0.0000
	0.10	1.0000±0.0000
	0.20	0.9931±0.0104
Bias drift (β)	0.10	1.0000±0.0000
	0.20	1.0000±0.0000
	0.50	0.9771±0.0000

## Data Availability

The data from the current study cannot be made publicly available due to confidentiality concerns.

## Appendix A. Implementation Details

### Appendix A.1. Hyperparameter Configuration

We evaluate hyperparameters over predefined ranges and select the final configuration based on the resulting threshold behavior. The hyperparameter configurations selected for all experiments are reported in Table A1 and Table A2.

**Table A1:** Hyperparameter configurations for threshold module.

Hyperparameter	Symbol	Range	Default
Drift detection 1	$\delta_{1}$	0.90–0.99	0.95
Drift detection 1	$\delta_{2}$	0.70–0.98	0.85
EWMA smoothing factor	α	0.7–1.0	0.90
Window length	*k*	50–500	100
Aggregation horizon	*m*	1–8	5
Threshold tolerance	ϵ	0.01–0.50	0.20
Drift threshold	$t_{p}$	0.5–0.9	0.9/1.1 of upper/lower threshold

**Table A2:** Model architecture of the lightweight forecasting decoder.

Hyperparameter	Value
Number of decoder layers (*N*)	1
Embedding dimension ($d_{\mathrm{model}}$)	32
Number of attention heads (*h*)	1
Attention head dimension ($d_{k}=d_{v}$)	32
Attention mechanism	Local causal self-attention
Local window size (*w*)	16
Feed-forward hidden dimension ($d_{\mathrm{ff}}$)	64
Activation function	GELU/ReLU

### Appendix A.2. Dataset Annotation and Data Splitting Protocol

The anomaly labels in the satellite thruster dataset are manually labeled based on engineering logs and expert knowledge. Specifically, abnormal operation intervals are identified and labeled by domain experts according to telemetry patterns and maintenance records. In addition to fault-related anomalies, thruster ignition events are also treated as detection targets, as they correspond to abrupt and structured changes in telemetry signals.

Regarding the split protocol, the dataset is split chronologically into training, validation, and test sets with a ratio of 60%/20%/20%.

## Appendix B. Additional Experiment Results

### Appendix B.1. Event-Based Evaluation

Following [43], the delay threshold *K* for the Yahoo dataset is set to 3, and for the thruster dataset, *K* is configured at 5. The result are shown in Table A3.

**Table A3:** Delayed F1-score on the Yahoo and Satellite datasets over 5 random seeds. **Bold** values indicate the best performance.

Method	Yahoo	Satellite
OCSVM	0.030±0.005	0.520±0.002
Isolation Forest	0.301±0.004	0.269±0.001
SPOT	0.417±0.002	0.610±0.003
DSPOT	0.322±0.007	0.659±0.003
SI-ATFD	0.385±0.004	0.616±0.004
AT2SD	0.442±0.010	0.582±0.006
DONUT	0.207±0.012	0.602±0.007
TranAD	0.452±0.004	0.759±0.005
Anomaly Transformer	0.274±0.001	0.745±0.004
Ours	0.512±0.002	0.873±0.000

### Appendix B.2. Statistical Test

**Table A4:** Paired *t*-test result for TranAD and our proposed method.

Dataset	F1 (*p*)	Delayed-F1 (*p*)
Yahoo	<0.001	<0.001
Satellite	<0.001	<0.001

### Appendix B.3. Ablation Results

**Table A5:** Quantitative ablation results on two datasets. ↑ indicates that higher values are better.

Method	Yahoo			Satellite		
	F1 ↑	Precision ↑	Recall ↑	F1 ↑	Precision ↑	Recall ↑
Full model	0.507	0.553	0.468	0.843	0.925	0.777
w/o TF	0.486	0.507	0.467	0.660	0.952	0.532
w/o Dy	0.217	0.786	0.126	0.743	0.946	0.612
w/o Fu	0.473	0.496	0.452	0.873	0.944	0.812
w/o TF + w/o Fu	0.424	0.433	0.416	0.800	0.864	0.745
